# Estimating the heritability of nitrogen and carbon isotopes in the tail hair of beef cattle

**DOI:** 10.1186/s12711-023-00870-7

**Published:** 2024-01-03

**Authors:** Morteza Moradi, Christie L. Warburton, Laercio Ribeiro Porto-Neto, Luis F. P. Silva

**Affiliations:** 1 Queensland Alliance for Agriculture and Food Innovation, The University of Queensland, Gatton, Qld 4343 Australia; 2 CSIRO Agriculture and Food, Queensland Bioscience Precinct, Brisbane, Qld 4067 Australia

## Abstract

**Background:**

The natural abundance of nitrogen (δ^15^N) and carbon (δ^13^C) isotopes in animal tissues are used to estimate an animal’s efficiency in nitrogen utilization, and their feed conversion efficiency, especially in tropical grazing systems with prolonged protein restriction. It is postulated that selection for improving these two characteristics (δ^15^N and δ^13^C) would assist the optimisation of the adaptation in ever-changing environments, particularly in response to climate change. The aim of this study was to determine the heritability of δ^15^N and δ^13^C in the tail hair of tropically adapted beef cattle to validate their inclusion in genetic breeding programs.

**Methods:**

In total, 492 steers from two breeds, Brahman (n = 268) and Droughtmaster (n = 224) were used in this study. These steers were managed in two mixed breed contemporary groups across two years (year of weaning): steers weaned in 2019 (n = 250) and 2020 (n = 242). Samples of tail switch hair representing hair segments grown during the dry season were collected and analysed for δ^15^N and δ^13^C with isotope-ratio mass spectrometry. Heritability and variance components were estimated in a univariate multibreed (and single breed) animal model in WOMBAT and ASReml using three generations of full pedigree.

**Results:**

The estimated heritability of both traits was significantly different from 0, i.e. 0.43 ± 0.14 and 0.41 ± 0.15 for δ^15^N and δ^13^C, respectively. These traits had favourable moderate to high genetic and phenotypic correlations (− 0.78 ± 0.16 and − 0.40 ± 0.04, respectively). The study also provides informative single-breed results in spite of the limited sample size, with estimated heritability values of 0.37 ± 0.19 and 0.19 ± 0.17 for δ^15^N and δ^13^C in Brahman, and 0.36 ± 0.21 and 0.46 ± 0.22 for δ^15^N and δ^13^C in Droughtmaster, respectively.

**Conclusions:**

The findings of this study show, for the first time, that the natural abundances of both nitrogen and carbon isotopes in the tail hair in cattle may be moderately heritable. With further research and validation, tail hair isotopes can become a practical tool for the large-scale selection of more efficient cattle.

**Supplementary Information:**

The online version contains supplementary material available at 10.1186/s12711-023-00870-7.

## Background

Over half of the world’s beef is produced in tropical and subtropical pastures, and production in these areas is growing faster than the global average [1, 2]. Approximately 70% of the projected increase in beef production that is required to meet rising demands by 2050 is anticipated to come from these regions [3, 4]. Tropical grazing systems are particularly influenced by seasonal variation in pasture quality and quantity [5], with a marked protein deficiency during the dry season [6]. In this situation, nitrogen recycling plays a key role in differences in feed efficiency between individuals [7]. Studies have shown that ruminants with better nitrogen use efficiency (NUE) have improved feed efficiency during growth [8, 9], better reproductive efficiency when raised in harsh environments [9], and better feed efficiency during lactation [10, 11]. In addition, improving the NUE of cattle can reduce total greenhouse gas emissions by reducing nitrogen excretion [12].

Because of the additive benefits of faster growth, more efficient use of available pastures and better reproductive performance, small increments in the efficiency of cattle to use available nutrients, can have a significant impact on the total profitability of grazing systems. Working in northern Australia, Ash et al. [13] calculated that a modest improvement of 3% in rumen efficiency (from 42 to 45% digestibility) would be sufficient to increase the net profit of cattle operations by 57%. Although clearly beneficial to cattle production, measuring NUE is not trivial, as it involves measuring (or correctly estimating) total nitrogen excretion in faeces and urine [14, 15]. It has recently been demonstrated that the natural abundance of the heavier ^15^N stable isotope (δ^15^N) in bovine tissues can be used as a proxy to estimate the NUE of individual animals [9, 14]. Lower δ^15^N in plasma or tail hair has been associated with better NUE and feed efficiency of steers and dairy cows [9]. δ^15^N can be measured in animal proteins, such as from tail hair, plasma, milk, and skeletal muscle [9, 14, 16, 17]. Although the correlation between δ^15^N and NUE has been established for individual animals, the heritability (*h*^2^) of this trait is still unknown [9, 14, 16, 17].

Apart from δ^15^N in body proteins, there is evidence that the natural abundance of the heavier ^13^C stable isotope (δ^13^C) might also be useful in explaining variations associated with intake and feed efficiency. In grazing systems, δ^13^C in ruminant tissues has been used to indicate individual differences in grazing behaviour and quantifying total legume intake [18] which, in turn, can affect feed efficiency and methane production [19, 20]. In addition, recent work with sheep has indicated the possibility to estimate feed efficiency based on changes in δ^13^C during a feeding trial [21].

Assuming a 12% increase in saleable live weight production because of the combined effect of better pregnancy rates and better growth efficiency of cattle with lower δ^15^N [9, 22], considering a population of 14.2 million cattle in northern Australia [23], and assuming 50% of producers implementing some form of genetic testing for breeding purposes, the potential economic value of using this new trait (δ^15^N) for the Australian beef cattle industry might be up to $3,935 million over 30 years or $51.73 per head/year. Therefore, the aim of this study was to determine if δ^15^N and δ^13^C are heritable in tropically adapted beef breeds, which, if it is the case, may merit their inclusion in north Australian breeding programs in the future to select for more feed efficient tropically adapted beef cattle.

## Methods

### Animals and contemporary groups

In total, 492 *Bos indicus* steers (268 Brahman and 224 Droughtmaster) were used in this study. The steers were part of BREEDPLAN, a beef cattle genetic evaluation system used in Australia [24] and had full sire and dam pedigree information. The steers are also part of the Repronomics project, which aims at collecting reproductive phenotypes and genotypes in northern Australia [25]. Briefly, sires used in this project were obtained from commercial studs and consisted of proven artificial insemination and naturally mated bulls as well as some unproven young sires [25]. Any sires that had limited or no daughters recorded for reproduction traits in BREEDPLAN were combined in the Repronomics project with the aim of producing 15 to 20 daughters per sire, to obtain daughter reproductive records.

For Droughtmasters, older sires were also selected for the estimation of breed genetic parameters [25]. All produced calves were recorded from birth to weaning for birth date, birth weight, weaning weight, and several other measurements [25]. The animals were born at the Spyglass Beef Research facility (Charters Towers, QLD) and transported to the Warraka station (Taroom, QLD) for backgrounding before being sent to a feedlot for finishing. At the Warraka station, the steers were kept on paddocks, which consisted mostly of Buffel grass (*Cenchrus ciliaris*) and Leucaena (*Leucaena leucocephala*).

**Fig. 1:**
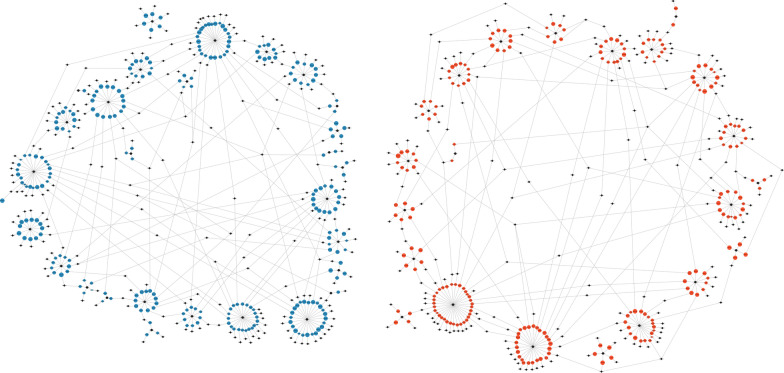
Pedigromics visualisation of one-generation pedigree focused on animals with traits, distributed as sire families. Sires are represented by black circles; dams and represented by black diamonds; and progeny with trait of interest (δ15N) are represented by blue circles (Brahman) or red circles (Droughtmaster) scaled by the δ^15^N values

The data were checked for the number of progenies per sire to ensure that the number of progenies generated per sire was similar between breeds and thus to avoid bias in the multibreed genomic evaluation. To visually inspect the relationships between animals, the network-based Pedigromics pipeline [26] was applied to a one-generation pedigree linked to animals with phenotypes (Fig. 1).

The sires used in this study were chosen to represent a wide range of genetics in the industry and sires were selected within breed as they have no recent genetic relationship with each other. The sire statistics are summarised in Table 1. Two mixed breed, contemporary groups of steers were used in this study: steers weaned in 2019 (n = 254) and steers weaned in 2020 (n = 243). Throughout the study, all the steers remained within their contemporary group, were weighed on the same day, and were subjected to the same management practices.

**Table 1:** Summary statistics of numbers of sires used per breed, average number of progenies per sire, minimum number of progenies per sire and maximum number of progenies per sire

Breed	Number sires	Average number progeny	Minimum number progeny	Maximum number progeny
Brahman	28	9.6	1	25
Droughtmaster	23	9.9	1	33

For the 2019 group, the animals were weighed three times: in April 2020 before transport to Warraka, in September 2020, and in May 2021 before being shipped to a feedlot. For the 2020 group, the steers were also weighed three times: in May 2020 before transport to Warraka, in November 2020, and in July 2021. Average daily gain (ADG) was calculated for the three periods, period 1 representing the ADG from birth until transport to Warraka, period 2, and period 3. The steers received a liquid urea and mineral supplement (Anipro Cattle, Performance Feeds Pty Ltd, Kingsthorpe, QLD, Australia) during the dry season.

### Sample collections

Samples of hair from the tail switch of the steers were collected in May 2021 for the 2019 group and in November 2021 for the 2020 group. The tail switch is a good source to estimate the long-period nutritional information of animals as they are the longest hair of cattle [27]. Segments of the tail hair were selected to represent hair growth during the dry season of the year, with poor forage quality, as nitrogen use efficiency (NUE) is better related to feed efficiency in protein-limiting diets [8, 22]. Based on rainfall data, and assuming that cattle hair grows on average 2 cm per month [28, 29], the segment of tail hair between 18 and19 cm from the bulb was used (representing hair growing in August 2020). For the 2020 group, the 1 cm of hair closest to the bulb was used, representing hair growing in October 2021.

There are advantages of using the tail hair instead of plasma or skeletal muscle for isotopic analyses. The isotopes of plasma or muscle show the information at the time when the sample is collected, so substantial variation could be observed. Using the tail hair can access the long-period nutritional history of the animals. In addition, sampling tail hair is easy and quick [27, 28, 30], non-invasive and with minimal animal disturbance [28], and can be stably stored at room temperature [30].

### Processing of tail hair

The tail hair samples were processed as described by Schwertl et al. [27]. Briefly, hair was cut at the desired segment, washed by ultra-sonication with deionised water (30 min), dried at 40 °C for 48 h, soaked in a 2:1 mixture of methanol: chloroform for 2 h, rinsed and soaked with deionised water for 30 min, rinsed and dried at 40 °C for 48 h. After processing, approximately 1 mg of tail hair was weighed into tin boats and submitted for isotope analysis at the Stable Isotope Geochemistry Laboratory at the University of Queensland, using an IsoPrime100 isotope-ratio mass spectrometry (Isoprime Ltd, Cheadle, UK) with dual inlet and coupled with a vario Pyro cube (Elementar Australia Pty, Sydney).

The stable isotope values are reported using the standard delta notation (δ per mil, ‰) calculated as follows: δX (‰) = [(R_sample_ – R_standard_)/R_standard_], where δX is δ^15^N or δ^13^C, and R is the ratio of the heavy to light stable isotope in the sample (^15^N/^14^N) or (^13^C/^12^C) and the standard (R_standard_). The results are reported against the AIR international and PDB standard for δ^15^N and δ^13^C, respectively.

### Statistical analysis

The data for 492 steers are summarized in Table 2. Data from the 2019 and 2020 steers were used for the estimation of the *h*^2^ of δ^15^N and δ^13^C in tail hair, using a three-generation pedigree.

**Table 2:** Descriptive statistics for steers weaned in 2019 or 2020

Item	Year	Breed							
		Brahman				Droughtmaster			
		Mean	Min	Max	SD	Mean	Min	Max	SD
Number of animals	2019	131				119			
δ^15^N (‰)		9.65	6.27	11.62	0.78	9.62	6.84	11.01	0.67
δ^13^C (‰)		− 12.51	− 16.82	− 10.52	1.15	− 12.29	− 17.1	− 10.6	0.99
ADG-P1^a^		0.46	0.32	0.59	0.05	0.49	0.31	0.66	0.58
ADG-P2^a^		0.65	0.30	0.86	0.11	0.66	0.32	1.05	0.12
ADG-P3^a^		0.71	0.50	0.96	0.79	0.72	0.52	0.93	0.07
Overall ADG^a^		0.56	0.45	0.69	0.04	0.58	0.47	0.68	0.04
Number of animals	2020	137				105			
δ^15^N (‰)		11.64	10.09	12.7	0.49	11.2	9.87	12.85	0.66
δ^13^C (‰)		− 15.97	− 18.62	− 13.06	1.00	− 15.53	− 17.9	− 12.81	1.17
ADG-P1^b^		0.81	0.53	1.12	0.11	0.83	0.54	1.23	0.14
ADG-P2^b^		0.39	0.20	0.64	0.07	0.42	0.22	0.59	0.08
ADG-P3^b^		0.62	0.44	0.78	0.07	0.61	0.43	0.85	0.07
Overall ADG^b^		0.61	0.50	0.76	0.05	0.61	0.51	0.79	0.05

δ^15^N, ratio of ^15^N:^14^N. δ^13^C, ratio of ^13^C:^12^C. ADG-P1^a^, (birth to Apr 2020, kg/d). ADG-P2^a^, (Apr to Sep 2020, kg/d). ADG-P3^a^, (Sep 2020 to May 2021, kg/d). Overall ADG^a^, (birth to May 2021, kg/d). ADG-P1^b^, (birth to May 2020, kg/d). ADG-P2^b^, (May to Nov 2020, kg/d). ADG-P3^b^, (Nov 2020 to July 2021, kg/d). Overall ADG^b^, (birth to July 2021, kg/d)

For the multibreed analysis, the significant fixed effects of the model were determined in RStudio (version 4.1.0) using linear regression. After significance testing, the significant fixed effects (P < 0.05) were fitted in a model using WOMBAT [31] for univariate analysis. The tested effects were age, breed, year (as contemporary group), ADG from birth to weaning and transport to backgrounding property (ADG-P1), ADG from weaning to the end of the dry season (ADG-P2), and ADG from the end of the dry season until the end of the backgrounding phase (ADG-P3). For the δ^15^N trait, ADG-P1, breed, and year (as contemporary groups) were significant and included in the model, and for the δ^13^C trait, ADG-P2, ADG-P3, age, breed, and year were included.

Although fitting ADG during different periods in the model removes some of the genetic variation that could be attributed to δ^15^N and δ^13^C, the fixed effects were included as a proxy for dietary changes in different seasons, as the diet quality affects the values of δ^15^N and δ^13^C in tail hairs. Single-breed models were the same as the multibreed models described above, but without the breed effect fitted in the model [31]. Univariate and bivariate analyses were performed using the single-breed and multibreed data (n = 492) for both δ^15^N and δ^13^C. Heritability and variance components were estimated in a univariate multibreed animal model in WOMBAT using three generations of available pedigree. Bivariate analyses were used in WOMBAT to estimate the genetic correlations between δ^15^N and δ^13^C in these multibreed cohorts.

In WOMBAT, approximate sampling errors of covariance components and genetic parameters are estimated using information derived from the likelihood function, with the inverse of the average matrix at the point of convergence [31]. When working with small populations, such as those in the current study, sampling errors can be underestimated [32]. For completeness, univariate and bivariate models for the single and multibreed analyses were also analysed using ASReml version 4.1 [33]. As both ASReml and WOMBAT presented similar results for the parameter estimates and their standard errors (see Additional file 1: Table S1, Additional file 2: Table S2 and Additional file 3: Table S3), only the results estimated with WOMBAT are presented.

Variance components were estimated using the model:

$$\mathbf{y}=\mathbf{X}\mathbf{b}+{\mathbf{Z}}_{\mathbf{A}}\mathbf{a}+\mathbf{e},$$

where $\mathbf{y}$ is the vector of observations of δ^15^N or δ^13^C, and $\mathbf{X}$ is the incidence matrix relating observations to fixed effects, ${\mathbf{Z}}_{\mathbf{A}}$ is then incidence matrix relating observations to direct genetic effects, $\mathbf{b}$ is the vector of fixed effects, **a** is the vector of direct genetic effects and $\mathbf{e}$ is the vector of residuals. Furthermore, $\text{var}\left(\mathbf{a}\right)=\mathbf{A}{{\upsigma }}_{a}^{2}$ and $\text{var}\left(\mathbf{e}\right)=\mathbf{I}{{\upsigma }}_{\text{e}}^{2}$ where $\mathbf{A}$ is the numerator relationship matrix, $\mathbf{I}$ is the identity matrix, ${{\upsigma }}_{a}^{2}$ is the direct additive genetic variance and ${{\upsigma }}_{\text{e}}^{2}$ is the residual error variance.

## Results

Our results indicate that both traits, δ^15^N and δ^13^C in the tail hair, are moderately heritable in a multibreed population of tropically adapted beef cattle, i.e. 0.43 (± 0.14) and 0.41 (± 0.15) for δ^15^N and δ^13^C, respectively (see Table 3). The small standard errors compared to the estimated heritabilities, suggest that these results were significantly different from 0.

**Table 3:** Estimated heritability (*h*^2^), genetic variance (${{\upsigma }}_{\text{A}}^{2}$), phenotypic variance (${{\upsigma }}_{\text{P}}^{2}$), and residual variance (${{\upsigma }}_{\mathbf{E}}^{2}$) of δ^15^N and δ^13^C in a multibreed population of Brahman and Droughtmaster steers (standard errors in parentheses)

Items	δ^15^N	δ^13^C
*h*^2^	0.43 (± 0.14)	0.41 (± 0.15)
${{\upsigma }}_{\text{A}}^{2}$	0.19 (± 0.07)	0.47 (± 0.18)
${{\upsigma }}_{\text{P}}^{2}$	0.44 (± 0.03)	1.14 (± 0.08)
${{\upsigma }}_{\text{E}}^{2}$	0.25 (± 0.06)	0.67 (± 0.15)

The bivariate analyses in the multibreed population show that, in spite of the strong genetic correlation between the two traits (− 0.78 ± 0.16), the phenotypic correlation between δ^15^N and δ^13^C in the tail hair was lower (− 0.40 ± 0.04). Furthermore, the negative correlation coefficients suggest the existence of a favourable genetic correlation between δ^15^N and δ^13^C, as lower δ^15^N and higher δ^13^C values infer more feed-efficient animals. The standard errors of both correlations confirm that the genetic and phenotypic correlations were significantly different from 0, which suggests that each trait exhibits some genetic control over the other.

**Fig. 2:**
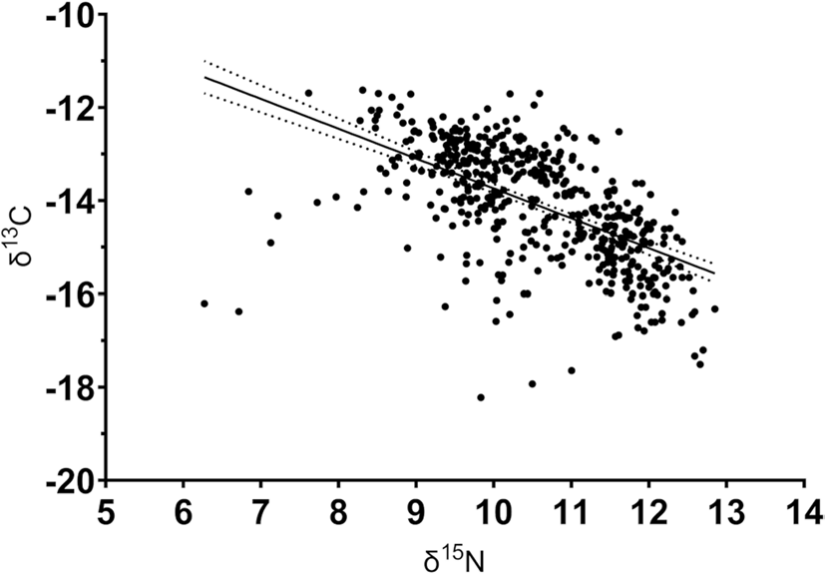
The cohort-adjusted relationship of the natural abundance of nitrogen (δ^15^N) and carbon (δ^13^C) isotopes in the tail hair of growing steers.  The regressions equation was δ^13^C = − 0.64 δ^15^N – 7.33, R^2^  = 0.35, P < 0.01

The existing variation in the studied population and the year-adjusted regression between δ^15^N and δ^13^C can be visualised in Fig. 2. The moderate negative relationship between the δ^15^N and δ^13^C in the tail hair was highly significant (P < 0.01, R^2^ = 0.35).

In spite of the limited number of animals for each breed, it is informative to present the single-breed results. For the Brahman breed, the estimated *h*^2^ was 0.37 ± 0.19 and 0.19 ± 0.17 for δ^15^N and δ^13^C, respectively; while for the Droughtmaster breed, *h*^2^ was 0.36 ± 0.21 and 0.46 0.22% for δ^15^N and δ^13^C, respectively (Table 4).

**Table 4:** Estimated genetic variance (σ^2^_A_), phenotypic variance (σ^2^_P_), residual variance (σ^2^_E_) and heritability (*h*^2^) of δ^15^N and δ^13^C estimated in a single breed analysis of Brahman and Droughtmaster steers (standard errors in parentheses)

Items	Brahman		Droughtmaster	
	δ^15^N	δ^13^C	δ^15^N	δ^13^C
*h*^2^	0.37 (± 0.19)	0.19 (± 0.17)	0.36 (± 0.21)	0.46 (± 0.22)
${{\upsigma }}_{\text{A}}^{2}$	0.16 (± 0.09)	0.20 (± 0.19)	0.16 (± 0.09)	0.54 (± 0.28)
${{\upsigma }}_{\text{P}}^{2}$	0.43 (± 0.04)	1.09 (± 0.10)	0.43 (± 0.04)	1.17 (± 0.12)
${{\upsigma }}_{\text{E}}^{2}$	0.27 (± 0.08)	0.89 (± 0.18)	0.28 (± 0.08)	0.63 (± 0.23)

The bivariate estimates for the single-breed analysis are in Table 5. The genetic and phenotypic correlations between δ^15^N and δ^13^C were estimated to be − 0.98 ± 0.44 and − 0.29 ± 0.06 for the Brahman, and − 0.85 ± 0.16, and − 0.52 ± 0.05 for the Droughtmaster breeds, respectively.

**Table 5:** Bivariate estimates for the phenotypic (r_P_) and genetic correlations (r_G_) between δ^15^N and δ^13^C for the two breeds (standard errors)

Items	Brahman	Droughtmaster
Phenotypic correlation (r_P_)	− 0.29 (± 0.06)	− 0.52 (± 0.05)
Genetic correlation (r_G_)	− 0.98 (± 0.44)	− 0.85 (± 0.16)

## Discussion

The results suggest that both traits, δ^15^N and δ^13^C, are heritable in tropically adapted beef cattle. Therefore, analysis of stable isotopes in the tail hair of cattle has the potential to be used as a selection trait in breeding programs. To the authors’ knowledge, this is the first study to estimate the heritability of these two traits δ^15^N and δ^13^C in cattle.

The association between δ^15^N in tail hair and nitrogen use efficiency (NUE) derives from the fact that the main form of metabolisable nitrogen loss, urea in urine, is depleted of ^15^N [34]. Therefore, bovine tissues, in general, are enriched for ^15^N compared to the diet [22, 34], and tissue proteins from more efficient cattle (less ^14^N being lost in the urine) will be less enriched in ^15^N than tissue proteins from less efficient cattle (more ^14^N being lost in the urine). In dairy cattle, NUE has been estimated based on the proportion of nitrogen intake that is excreted in milk, without direct measurements of total faeces and urine production, and *h*^2^ of NUE for dairy cattle has been estimated to be 0.11 ± 0.08 [35] or 0.13 ± 0.019 [36].

Our results demonstrate that the heritability of δ^15^N in tail hair is of greater or similar magnitude than that of other important traits commonly used in beef genetic programmes. For example, *h*^2^ has been estimated to be 0.10 for fertility, 0.24 for weaning weight, 0.14 for 200-day weight, 0.26 for 400-day weight, 0.48 for 600-day weight, 0.33 for ADG, and 0.20 for feed conversion ratio [37, 38]. Thus, by considering the large range of δ^15^N values among individuals in the present study (6.27 to 11.62‰, and 9.87 to 12.85‰, for 2019 and 2020 groups, respectively) and the estimated *h*^2^, it is expected that genetic gains can be made when using tail hair δ^15^N for selecting cattle [38]. Nonetheless, the use of tail hair δ^15^N as a proxy for NUE needs to be further validated across diverse breeds and environments before it can be applied to large-scale selection of cattle.

The enrichment of cattle tissue with ^13^C has also been used to improve the information about dietary habits and feed efficiency. Although not previously related to NUE, the individual variation in δ^13^C can indicate the proportion of legume intake in mixed pastures and different intakes of supplements. Plants with different photosynthetic pathways (legumes vs. tropical grasses, temperate grasses vs. tropical grasses) will have markedly different δ^13^C signatures leading to differences in δ^13^C in animal tissues [16, 18, 28]. In addition, it has recently been demonstrated that the rate of δ^13^C incorporation in sheep tissues after a diet change was strongly related to feed efficiency, as sheep with high maintenance requirements likely deposited more ^13^C after the dietary change [21].

In the present study, we found a favourable moderate to strong phenotypic and genetic correlation between δ^15^N and δ^13^C in tail hair. As for the N isotopes, the urine of cattle has been shown to have a different ^12^C:^13^C ratio than the diet. However, in contrast to the N isotopes, the urine of cattle that eat forage-based diets is more enriched in the heavy isotope (^13^C) than the diet, creating a difference in the isotopic fractionation between individuals [16]. Thus, as the urine of cattle is less enriched in ^15^N and more enriched in ^13^C, this would explain the negative, and favourable, genetic correlation between both traits.

It is important to highlight that the tail hair was collected during the driest part of the year, when the diet has a low protein content, to allow the animals to express differences in nitrogen recycling and nitrogen conservation mechanisms. The correlation between both traits could be very different if measured in better quality diets. Nonetheless, the results suggest that the genes that control δ^15^N and δ^13^C in the tail hair are linked; therefore, selection for the δ^15^N trait will also have a desirable influence on δ^13^C.

The present study was conducted on a single farm over 2 years and evaluated only two breeds, Brahman and Droughtmaster. In spite of the limitations intrinsic to the small population size, single breed variance component estimates for δ^15^N had similar trends to the multibreed population. However, the variance component estimates for δ^13^C between the two breeds were different. While these estimates for Droughtmasters were similar to multibreed results, for Brahman steers, the *h*^2^ estimates were lower. This agrees with Johnston et al. [39] in that the variance components for some traits can be very different between Brahman and tropical composite beef breeds. Nonetheless, the single-breed results suggest that these traits are heritable across both breeds and selection for δ^15^N and δ^13^C will result in desirable genetic progress.

## Conclusions

Our results provide evidence supporting the hypothesis that δ^15^N and δ^13^C may be heritable in tropically adapted beef cattle. Nonetheless, large population studies are needed to firmly establish the heritability of these new traits. In the multibreed population, the heritability of δ^15^N and δ^13^C in tail hair is estimated to be 0.43 and 0.41, respectively. These two traits have favourable moderate to strong genetic and phenotype correlations. To the authors’ knowledge, this is the first study to estimate the heritability of these traits in cattle, which further supports the use of the natural abundance of nitrogen and carbon isotopes to improve the nitrogen use efficiency of cattle.

### Supplementary information

**Additional file 1.**  Estimated genetic variance (σ^2^A ), phenotypic variance (σ^2^P ), residual variance (σ^2^E ) and heritability (*h*^2^) of δ^15^N and δ^13^C estimated in a multibreed population of Brahman and Droughtmaster steers using ASReml (standard errors in parentheses).

**Additional file 2.**  Estimated genetic variance (σ^2^A ), phenotypic variance (σ^2^P ), residual variance (σ^2^E ) and heritability (*h*^2^) of δ^15^N and δ^13^C estimated in a single breed analysis of Brahman and Droughtmaster steers using ASReml (standard errors in parentheses).

**Additional file 3.**  Bivariate estimates (correlations between δ^15^N and δ^13^C) for phenotypic correlation (r_P_ ) and genetic correlation (r_G_ ) for the two breeds estimated in ASReml (standard errors in parentheses).

## Data Availability

The datasets used during the current study are available from the corresponding author on reasonable request.

## References

[1] Cooke RF, Daigle CL, Moriel P, Smith SB, Tedeschi LO, Vendramini JM (2020). Cattle adapted to tropical and subtropical environments: social, nutritional, and carcass quality considerations. J Anim Sci.

[2] Hernandez A, Galina CS, Geffroy M, Jung J, Westin R, Berg C (2022). Cattle welfare aspects of production systems in the tropics. Anim Prod Sci.

[3] FAO (2009). How to feed the world in 2050.

[4] OECD/FAO. OECD-FAO Agricultural. Outlook 2020–2029. Paris: OECD Publishing and Rome: Food and Agriculture Organization. 2020.

[5] Machado L, Kinley RD, Magnusson M, de Nys R, Tomkins NW (2015). The potential of macroalgae for beef production systems in Northern Australia. J Appl Phycol.

[6] Detmann E, Paulino MF, de Campos Valadares Filho S, Huhtanen P (2014). Nutritional aspects applied to grazing cattle in the tropics: a review based on Brazilian results. Semina: Ciênc Agrár.

[7] Silva LFP, Dixon RM, Costa DFA (2019). Nitrogen recycling and feed efficiency of cattle fed protein-restricted diets. Anim Prod Sci.

[8] Carmona P, Costa DFA, Silva LFP (2020). Feed efficiency and nitrogen use rankings of Bos indicus steers differ on low and high protein diets. Anim Feed Sci Tech.

[9] Silva LFP, Hegarty RS, Meale SJ, Costa DAF, Fletcher MT (2022). Using the natural abundance of nitrogen isotopes to identify cattle with greater efficiency in protein-limiting diets. Animal.

[10] Cantalapiedra-Hijar G, Fouillet H, Huneau J-F, Fanchone A, Doreau M, Noziere P (2016). Relationship between efficiency of nitrogen utilization and isotopic nitrogen fractionation in dairy cows: contribution of digestion v. metabolism. Animal.

[11] Correa-Luna M, Johansen M, Noziere P, Chantelauze C, Nasrollahi S, Lund P (2022). Nitrogen isotopic discrimination as a biomarker of between-cow variation in the efficiency of nitrogen utilization for milk production: a meta-analysis. J Dairy Sci.

[12] Rotz CA, Asem-Hiablie S, Place S, Thoma G (2019). Environmental footprints of beef cattle production in the United States. Agric Syst.

[13] Ash A, Hunt L, McDonald C, Scanlan J, Bell L, Cowley R (2015). Boosting the productivity and profitability of northern Australian beef enterprises: exploring innovation options using simulation modelling and systems analysis. Agric Syst.

[14] Cantalapiedra-Hijar G, Ortigues-Marty I, Sepchat B, Agabriel J, Huneau JF, Fouillet H (2015). Diet–animal fractionation of nitrogen stable isotopes reflects the efficiency of nitrogen assimilation in ruminants. Br J Nutr.

[15] Asher A, Shabtay A, Cohen-Zinder M, Aharoni Y, Miron J, Agmon R (2018). Consistency of feed efficiency ranking and mechanisms associated with inter-animal variation among growing calves. J Anim Sci.

[16] Knobbe N, Vogl J, Pritzkow W, Panne U, Fry H, Lochotzke HM (2006). C and N stable isotope variation in urine and milk of cattle depending on the diet. Anal Bioanal Chem..

[17] Devincenzi T, Delfosse O, Andueza D, Nabinger C, Prache S (2014). Dose-dependent response of nitrogen stable isotope ratio to proportion of legumes in diet to authenticate lamb meat produced from legume-rich diets. Food Chem.

[18] Minson DJ, Ludlow MM, Troughton JH (1975). Differences in natural carbon isotope ratios of milk and hair from cattle grazing tropical and temperate pastures. Nature.

[19] Archimède H, Eugène M, Marie-Magdeleine C, Boval M, Martin C, Morgavi D (2011). Comparison of methane production between C3 and C4 grasses and legumes. Anim Feed Sci Tech.

[20] Sauvant D, Giger-Reverdin S (2009). Modelling of digestive interactions and methane production in ruminants. INRA Prod Anim.

[21] Dvergedal H, Kidane A, Klemetsdal G, Mydland LT, Øverland M, Olsen HF (2020). Individual phenotyping of feed efficiency in lambs fed stable isotopes through maize silage. Livest Sci.

[22] Guarnido-Lopez P, Ortigues-Marty I, Taussat S, Fossaert C, Renand G, Cantalapiedra-Hijar G (2021). Plasma proteins δ15N vs plasma urea as candidate biomarkers of between-animal variations of feed efficiency in beef cattle: phenotypic and genetic evaluation. Animal.

[23] Australian Bureau of Statistics. Agricultural Commodities, Australie. https://www.abs.gov.au/statistics/industry/agriculture/agricultural-commodities-australia/2021-22/ . Accessed 28 Nov 2023.

[24] Graser H, Tier B, Johnston D, Barwick S (2005). Genetic evaluation for the beef industry in Australia. Aust J Exp Agric.

[25] Johnston D, Grant T, Schatz T, Burns B, Fordyce G, Lyons R (2017). The repronomics project–enabling genetic improvement in reproduction in northern Australia. Proc Assoc Advmt Anim Breed Genet.

[26] Reverter-Gomez A, Dominik S, Ferraz J, Corrigan L, Porto-Neto L (2019). Pedigromics: a network-inspired approach to visualise and analyse pedigree structures. Proc Assoc Advmt Anim Breed Genet..

[27] Schwertl M, Auerswald K, Schnyder H (2003). Reconstruction of the isotopic history of animal diets by hair segmental analysis. Rapid Commun Mass Spectrom.

[28] Hammes V, Nüsse O, Isselstein J, Kayser M (2017). Using 13 C in cattle hair to trace back the maize level in the feeding regime—a field test. PLoS One..

[29] Cantalapiedra-Hijar G, Dewhurst RJ, Cheng L, Cabrita ARJ, Fonseca AJM, Noziere P (2018). Nitrogen isotopic fractionation as a biomarker for nitrogen use efficiency in ruminants: a meta-analysis. Animal.

[30] Tallo-Parra O, Albanell E, Carbajal A, Monclús L, Manteca X, Lopez-Bejar M (2017). Prediction of cortisol and progesterone concentrations in cow hair using near-infrared reflectance spectroscopy (NIRS). Appl Spectrosc.

[31] Meyer K (2007). WOMBAT—a tool for mixed model analyses in quantitative genetics by restricted maximum likelihood (REML). J Zhejiang Univ-Sci B.

[32] Visscher PM (1998). On the sampling variance of intraclass correlations and genetic correlations. Genetics.

[33] Gilmour A, Gogel B, Cullis B, Welham S, Thompson R (2015). ASReml user guide release 4.1 structural specification.

[34] Gannes LZ, Del Rio CM, Koch P (1998). Natural abundance variations in stable isotopes and their potential uses in animal physiological ecology. Comp Biochem Physiol A Mol Integr Physiol.

[35] Lopez-Villalobos N, Correa-Luna M, Burke J, Sneddon N, Schutz M, Donaghy D (2018). Genetic parameters for milk urea concentration and milk traits in New Zealand grazing dairy cattle. NZ J Anim Sci Prod.

[36] Chen Y, Vanderick S, Mota R, Grelet C, Gengler N, Consortium GplusE, Gengler N (2021). Estimation of genetic parameters for predicted nitrogen use efficiency and losses in early lactation of Holstein cows. J Dairy Sci..

[37] Torres-Vázquez JA, van der Werf JH, Clark SA (2018). Genetic and phenotypic associations of feed efficiency with growth and carcass traits in Australian Angus cattle. J Anim Sci.

[38] Massey JW, Vogt DW. Heritability and its use in animal breeding.https://extension.missouri.edu/publications/g2910. Accessed 21 May 2023.

[39] Johnston D, Barwick S, Corbet N, Fordyce G, Holroyd R, Williams PJ (2009). Genetics of heifer puberty in two tropical beef genotypes in northern Australia and associations with heifer-and steer-production traits. Anim Prod Sci.

