# Supplementary material for: Estimating the heritability of nitrogen and carbon isotopes in the tail hair of beef cattle
Source: Genet Sel Evol. 2024 Jan 3;56:3. doi: 10.1186/s12711-023-00870-7 (PMC10763070; doi:10.1186/s12711-023-00870-7)
Supplement: Supplementary file 1 — Additional file 1. Estimated genetic variance (σ2A ), phenotypic variance (σ2P ), residual variance (σ2E ) and heritability (h2) of δ15N and δ13C estimated in a multibreed population of Brahman and Droughtmaster steers using ASReml (standard errors in parentheses). [file 12711_2023_870_MOESM1_ESM.docx]

**Table S1.** Estimated genetic variance (σ^2^_A_), phenotypic variance (σ^2^_P_), residual variance (σ^2^_E_) and heritability (*h*^2^) of δ^15^N and δ^13^C estimated in a multibreed population of Brahman and Droughtmaster steers using ASReml (standard errors in parentheses).

| **Items** | **δ^15^N** | **δ^13^C** |
| --- | --- | --- |
| *h*^2^ | 0.42 (± 0.14) | 0.36 (± 0.18) |
| σ^2^_A_ | 0.19 (± 0.07) | 0.39 (± 0.20) |
| σ^2^_P_ | 0.45 (± 0.03) | 1.07 (± 0.09) |
| σ^2^_E_ | 0.26 (± 0.06) | 0.68 (± 0.18) |
