# Supplementary material for: Estimating the heritability of nitrogen and carbon isotopes in the tail hair of beef cattle
Source: Genet Sel Evol. 2024 Jan 3;56:3. doi: 10.1186/s12711-023-00870-7 (PMC10763070; doi:10.1186/s12711-023-00870-7)
Supplement: Supplementary file 2 — Additional file 2. Estimated genetic variance (σ2A ), phenotypic variance (σ2P ), residual variance (σ2E ) and heritability (h2) of δ15N and δ13C estimated in a single breed analysis of Brahman and Droughtmaster steers using ASReml (standard errors in parentheses). [file 12711_2023_870_MOESM2_ESM.docx]

**Table S2**. Estimated genetic variance (σ^2^_A_), phenotypic variance (σ^2^_P_), residual variance (σ^2^_E_) and heritability (*h*^2^) of δ^15^N and δ^13^C estimated in a single breed analysis of Brahman and Droughtmaster steers using ASReml (standard errors in parentheses)

| **Items** | **Brahman** | | **Droughtmaster** | |
| --- | --- | --- | --- | --- |
|  | **δ^15^N** | **δ^13^C** | **δ^15^N** | **δ^13^C** |
| *h*^2^ | 0.38 (± 0.19) | 0.20 (± 0.26) | 0.33 (± 0.20) | 0.79 (± 0.39)^*^ |
| σ^2^_A_ | 0.16 (± 0.09) | 0.20 (± 0.26) | 0.16 (± 0.10) | 0.93 (± 0.54)^*^ |
| σ^2^_P_ | 0.43 (± 0.03) | 0.99 (± 0.17) | 0.46 (± 0.05) | 1.17 (± 0.18)^*^ |
| σ^2^_E_ | 0.27 (± 0.08) | 0.79 (± 0.25) | 0.31 (± 0.09) | 0.25 (± 0.45)^*^ |

^*^Log likelihood converged but parameter estimates did not converge
