# Supplementary material for: Estimating the heritability of nitrogen and carbon isotopes in the tail hair of beef cattle
Source: Genet Sel Evol. 2024 Jan 3;56:3. doi: 10.1186/s12711-023-00870-7 (PMC10763070; doi:10.1186/s12711-023-00870-7)
Supplement: Supplementary file 3 — Additional file 3. Bivariate estimates (correlations between δ15N and δ13C) for phenotypic correlation (rP ) and genetic correlation (rG ) for the two breeds estimated in ASReml (standard errors in parentheses). [file 12711_2023_870_MOESM3_ESM.docx]

**Table S3**. Bivariate estimates (correlations between δ^15^N and δ^13^C) for phenotypic correlation (r_P_) and genetic correlation (r_G_) for the two breeds estimated in ASReml (standard errors in parentheses)

| **Items** | **Brahman** | **Droughtmaster** | **Multibreeed** |
| --- | --- | --- | --- |
| Phenotypic correlation (r_P_) | -0.37 (± 0.07) | -0.59 (± 0.06) | -0.44 (± 0.05) |
| Genetic correlation (r_G_) | -0.83 (± 0.45) | -0.86 (± 0.21) | -0.81 (± 0.20) |
